# Our Journal

**Published:** 1851-01

**Authors:** 


ARTICLE II.

### OUR JOURNAL.

We have often been solicited to publish the Journal monthly, instead of only once in two months, and out of deference to the judgment of our friends in regard to its propriety, we have had the matter under serious advisement. But fearing that the increased expense might not be met by any increased punctuality on the part of our patrons in making prompt payment, we have not thought it best to try it, for however the circulation might be extended, it is but too manifest that without prompt payment, we would be greatly the loosers by the change.

We however, propose to make the change gradually, by commencing on the first of April next, the issue of the North Western Medical Intelligencer, and to continue it every other month, designed to keep our readers informed in reference to all of the current medical news of the months intermediate between the issue of the Journal.

The intelligencer will contain sixteen pages, uniform with the Journal, but will make a separate volume, and will be sent to those of the subscribers to the Journal only who have paid all arrearages and advanced the money for the volume. We shall, with each issue, print only enough copies to supply such portion of our subscribers as have paid up at the time, and therefore we will not be able to supply any back numbers of the Intelligencer. It will not be sent to non-subscribers of the Journal at all.

The Journal in the mean time, will be issued regularly, as heretofore, giving about five hundred pages of reading matter for the same price. Thus those of our readers who pay in advance will get about 600 pages of reading matter for the same money, and be more regularly advised of passing events.

